# Effect of Mechanical Microenvironment on Collagen Self-Assembly In Vitro

**DOI:** 10.3390/jfb14040235

**Published:** 2023-04-21

**Authors:** Leihan Han, Jiexiang Lin, Chengfei Du, Chunqiu Zhang, Xin Wang, Qijin Feng

**Affiliations:** 1 Tianjin Key Laboratory for Advanced Mechatronic System Design and Intelligent Control, School of Mechanical Engineering, Tianjin University of Technology, Tianjin 300384, China; 13805415582@163.com (L.H.); linjiexiang@outlook.com (J.L.); ddccffb31@hotmail.com (C.D.); zhang_chunqiu@126.com (C.Z.); 2 National Demonstration Center for Experimental Mechanical and Electrical Engineering Education, Tianjin 300384, China; 3 Tianjin University of Traditional Chinese Medicine Second Affiliated Hospital, Tianjin 300151, China

**Keywords:** collagen, mechanical microenvironment, hyaluronic acid, atomic force microscope, self-assembly

## Abstract

Collagen, as a structural protein, is widely distributed in the human body. Many factors influence collagen self-assembly in vitro, including physical-chemical conditions and mechanical microenvironment, and play a key role in driving the structure and arrangement. However, the exact mechanism is unknown. The purpose of this paper is to investigate the changes in the structure and morphology of collagen self-assembly in vitro under mechanical microenvironment, as well as the critical role of hyaluronic acid in this process. Using bovine type I collagen as the research object, collagen solution is loaded into tensile and stress-strain gradient devices. The morphology and distribution of collagen is observed using an atomic force microscope while changing the concentration of collagen solution, mechanical loading strength, tensile speed, and ratio of collagen to hyaluronic acid. The results demonstrate that the mechanics field governs collagen fibers and changes their orientation. Stress magnifies the differences in results caused by different stress concentrations and sizes, and hyaluronic acid improves collagen fiber orientation. This research is critical for expanding the use of collagen-based biomaterials in tissue engineering.

## 1. Introduction

**Figure 1:**
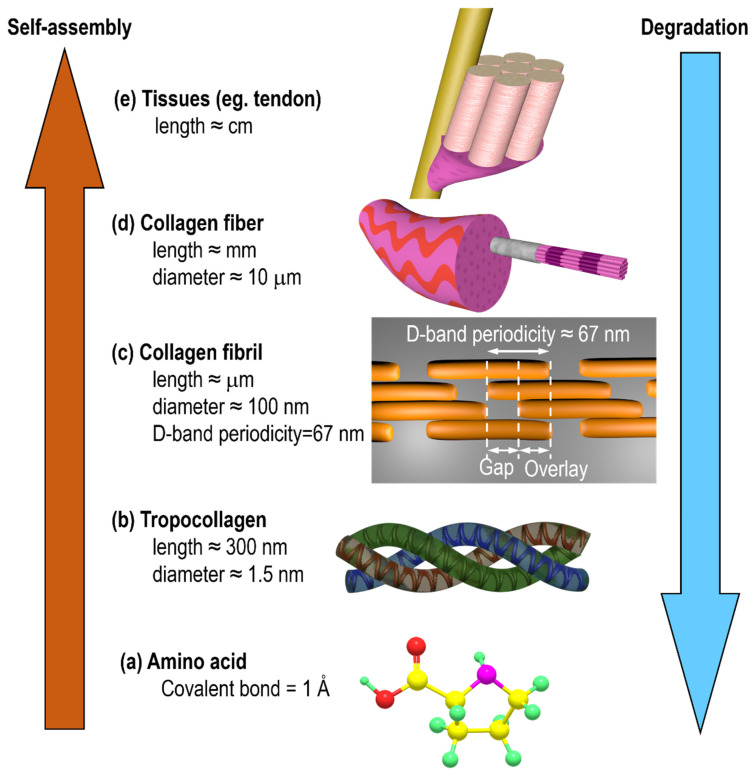
Schematic diagram of collagen hierarchical self-assembly from molecular to tissue in macroscale.

Collagen, the most common structural protein in humans and other vertebrates, is an essential component of human cornea, tendon, skin, bone, and cartilage, providing mechanical stability, elasticity, and strength to organisms [1,2,3,4,5]. At present, 29 kinds of collagen are known, among which type I collagen is the most widely studied [6,7]. Collagen self-assembly is the complex layered process from molecular to macroscale length [1,2]. Each collagen molecule, which is made up of three mutually helical peptide chains, is about 300 nm in length and 1.5 nm in diameter. Collagen molecules form fibers in a longitudinal staggered arrangement in their natural state, with a typical D-band periodicity (about 67 nm). The graded structure and D-band of collagen are shown in Figure 1.

Some changes in physical and chemical conditions affect the orientation and conformation of collagen. The concentration mainly affects the diameter, width, and roughness of self-assembled collagen. In 2007, Raspanti [8] conducted in vitro self-assembly experiments with three different collagen concentrations. The diameter of collagen fibers with an initial concentration of 0.5 mg/mL was found to be significantly larger than 1 mg/mL, while a massive and irregular superfiber was observed at a very low concentration of procollagen 0.1 mg/mL. This is made up of a transverse combination of smaller fibers and a surface with a distinct crimped structure. In 2017, Song [9] discovered that collagen self-assembled into small parallel fibers at low concentrations. The width of collagen fibers decreased substantially as collagen concentration increased, and the parallel collagen fibers were cross-linked by some fine fibers to form a reticular self-assembly structure. In 2013, Stylianou [10] used AFM to characterize collagen films formed on the surfaces of glass, mica, and polystyrene latex particles. The collagen membrane forms non-oriented fibers on the glass. The film formed on mica at the same speed consists of randomly oriented fibers and twisted fibers with a diameter of 500 nm to 2 mm, meaning the range of fiber diameters is wider. The collagen membrane formed on the surface of polystyrene has no vacancies or cracks, and its surface shows hexagonal collagen accumulation.

The effect of mechanics on biomaterials is currently being researched extensively [11,12,13,14], mainly in collagen orientation [13], and promotes collagen self-assembly [15]. Directional arrangement of collagen fibers is crucial for human life activities [14]. It can not only promote bone growth but also induce cells to secrete extracellular matrix with a directional structure [16]. People have also realized the significance of collagen’s directional arrangement [17]. Many experts from various fields have focused on the tissue engineering application of oriented collagen arrangement in recent years [17,18,19,20]. Currently, many systems are designed to align collagen during polymerization by applying tension to it. The main methods to induce the directional arrangement of collagen fibers are pre-stretching the polydimethylsiloxane (PDMS) [21] and stretching glass needle [15]. Pins [22] stretched collagen fibers in their uncrosslinked state. Stretching about 30% of uncrosslinked collagen fibers not only reduces their diameter and increases their tensile strength, but it also causes an abnormal failure mechanism that prevents the fiber from spreading a crack. Wilson [23] used an AFM probe to immobilize procollagen solution on a gold substrate in 2001 to draw a line in a specific direction. The collagen vulcanized, allowing it to securely adhere to the matrix. The solution attached to the gold surface pulls the collagen monomer onto the probe via cohesion and Coulomb force as the probe moves, assembling the collagen monomer into directional fibers. Paten [15] used glass needles to unidirectionally pull procollagen solution to prepare directional fibers in 2016. Pull the microneedle slowly out of the droplet. A continuous liquid column is formed between the micropipette and the droplet during the stretching process, in which collagen monomers are assembled into collagen fibers arranged along the direction of force, but the fiber orientation is not uniformly distributed. The fibers at the liquid column’s boundary are oriented along the stress direction, and the interior of the liquid column is made up of non-oriented short fibers. In 2020, Krashn [24] found collagen fibers were not delaminated and were damaged little after the middle- and high-frequency stretching cycles, whereas the collagen fibers were damaged by sliding and delamination at low frequency, which caused the most damage to collagen fibers. Cross-linking in collagen fibers is critical for improving mechanical strength [25]. Cross-linking is critical in the development of an interconnected fiber material with adjustable toughness and strength [26]. Cross-linking prevents the slip of collagen molecules, whereas a lack of cross-linking weakens the structure [27]. The fiber becomes stronger as the cross-linking density increases, exhibiting increasingly brittle deformation characteristics [25]. In a nutshell, the density, type, and mechanical properties of cross-linking all impact the mechanical response of the fiber [26]. HA is an acidic mucopolysaccharide that can be used to cross-link collagen. In 2005, N. Davidenko [28] created collagen–HA scaffolds. Previous studies have only studied the effects of single forces or physical and chemical conditions on collagen self-assembly. There have been few studies on the effects of concentration and drawing speed on self-assembled collagen fibers under tension and stress-strain gradient changes. It is insufficient for the investigation into the effects of physical and chemical conditions on collagen self-assembly in a mechanical microenvironment. This study focuses on the distribution and structure of self-assembled collagen fibers in the stress-strain gradient field as a function of collagen solution concentration, stress intensity, and addition of various hyaluronic acid (HA) fractions. This work is mainly observed by AFM, a Scanning Electron Microscope (SEM), and a Transmission Electron Microscope (TEM). This study can further understand the self-assembly of collagen fibers in vitro and the application of collagen tissue engineering.

## 2. Methods and Materials

In this experiment, bovine type I collagen (Advanced Biomatrix, San Diego, CA, USA) is the main material. The substrate of the mechanical loader was a membrane made of PDMS membrane. The stretched collagen fibers were observed by light microscope (Axio Observer, ZEISS, Oberkochen, Germany). The mode of AFM (Bruker, Madison, WI, USA) scanning is Peakforce-QNM (Quantitative Nanomechanical Mapping), and liquid scanning is used. The probe used for scanning is Scanansys-Fluid (Bruker, Madison, WI, USA) and the size of scanning area is 10 μm × 10 μm, using SEM (FEI, Hillsboro, OR, USA) and TEM (FEI, Hillsboro, OR, USA) to assist with observation.

### 2.1. PDMS Membrane Preparation

The first step is to prepare PDMS culture membrane (polydimethylsiloxane, Dow Corning DC184), take 0.45 g prepolymer A into 60 mm^2^ petri dish, then add 0.05 g curing agent B according to the cross-linking ratio at 10:1, stir fully and put it on the vacuum rotary coating machine (SLK-O3000-S, SCILOGEX) to make it spread evenly. Then, put it into the vacuum pump to remove the bubbles caused by stirring in the PDMS glue, then sit horizontally for a period of time and bake in an electronic constant temperature drying oven (202-OAB, Tianjin Tester instrument) for 8 h. After baking, wash the sample in ultrasonic cleaner for 5 min in ultrasonic cleaning instrument (SB-100DT, Xinzhi Biotechnology), and then washed in plasma cleaning machine (TS-PL02, Dongxin Hi-Tech equipment) for 120 s.

### 2.2. Collagen Fibers Formed on the Stretching Device

**Figure 2:**
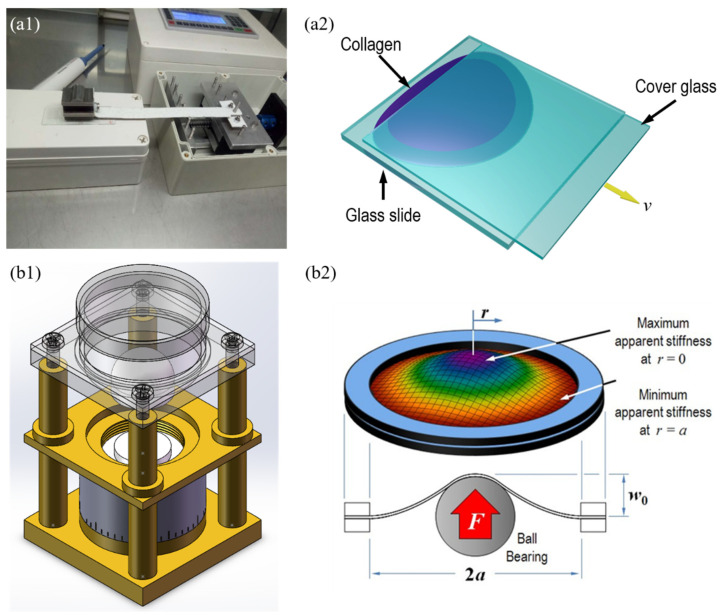
Experimental equipment. (**a1**) 2D stretching loading device; (**a2**) Schematic diagram of 2D stretching process; (**b1**) 3D Stress-strain gradient mechanical loading device; (**b2**) Schematic diagram of 3D stress gradient during loading.

The bovine type I collagen utilized in this experiment comes in three distinct concentrations: 1 mg/mL, 3 mg/mL, and 6 mg/mL (Type I bovine collagen solution, Advanced Biomatrix, New York, NY, USA). Collagen solution has a pH of 7.4. To make sure the collagen was entirely formed into collagen fibers after stretching, the collagen solution was incubated at 26 °C for 24 h. Figure 2(a1) is an experimental diagram, Figure 2(a2) is a schematic diagram. In order to minimize the impact of siphon on the experimental results, a rectangular iron block is also added to the upper end of the cover to ensure close contact between the glass sheets. The pipette gun absorbs 15 mL of the fiber solution before dripping it along the end of the cover slide to the overlap. The experiment involved totally submerging the overlapping portion of the covered glass in the fiber solution. The fiber solution is then pulled at various speeds (50 μm/s, 250 μm/s, 500 μm/s) by modifying the loading device. The whole pulling process takes less than 2 min (the cover plate moves about 18 mm). At the end of the experiment, the stretched fiber solution was dried and stored at room temperature for inspection.

### 2.3. Observation of Collagen Fibers Formed on Muscovite by SEM

Fresh mica tablets were placed in a 24-well plate and dripped with 300 μL collagen solution. The 24-well plate was cultured in a 37 °C cell incubator for 12 h, then the mica tablets were removed with tweezers and washed with Phosphate Buffered Saline (PBS, Solarbio) for 1 to 3 times. The 5 min was fixed with a fixed solution (2.5%, Solarbio) and cleaned. The mica tablets were then taken out and dehydrated in alcohol containing 25%, 50%, 75%, 90%, and 100%, respectively. Use the Critical Point Dryer (CPD, samdri-795, USA) to dry the sample. The mica sheet is glued to the SEM sample table with a conductive adhesive. Then, the sample will be gilded for 5 min with a gold-plated instrument (JFC-1600, Jieou Road Science and Trade) and scanned by SEM.

### 2.4. Observation of Collagen Fibers Formed in Carbon Network by TEM

The carbon net (FCFTH200-AU, Electron Microscope Sciences) was washed in a plasma cleaner for 15 s, and the collagen solution of 50 mL was dripped upward into the Petri dish lid to suspend the collagen solution so that the gold grid was suspended at the bottom of the droplet. After being cultured in a 37 °C cell incubator for 12 h, it was stained with Uranium Acetate Dye (Sigma-Aldrich, Saint Louis, MO, USA), washed with PBS, and cleaned with clean water 3 times, dried with filter paper, and observed in TEM.

### 2.5. AFM Observation of Collagen Fibers Formed under Mechanical Loading Device

The mechanical loading device is shown in Figure 2(b1), Figure 2(b2) is a stress diagram in loading. First, disassemble the mechanical loading device, cut it to a suitable size with aseptic scissors and install it on the mechanical loading device. The culture membrane is raised to a certain height with a spherical pressure head. The radial force and circumferential force decrease successively from the center to the edge of the membrane, and the prepared collagen solution is evenly added to the culture membrane of the mechanical loading device. The Petri dish containing PDMS membrane was used in the control group, and collagen solution was also added evenly. They were cultured in a 37 °C incubator for 12 h. After culture, the 5 min was fixed with special fixed liquid for PBS, then the mechanical loading device was reset, the culture membrane was removed, and three observation points were taken from the center to the edge of the membrane. All the unspecified observation points are the central points of the membrane. The culture membrane was cut to a suitable size with aseptic scissors and placed in a 35 mm^2^ Petri dish, added 3 mL PBS solution to the AFM sample table, and measured by AFM. In the control trial, three locations were randomly selected and measured by AFM. When the PDMS film is loaded in the mechanical loading device, the circumferential stress and circumferential stress decrease successively from high to low (from the center to the edge of the film).

## 3. Results and Discussion

### 3.1. Observation of Collagen Fibers Formed on the Stretching Device under Optical Microscope

**Figure 3:**
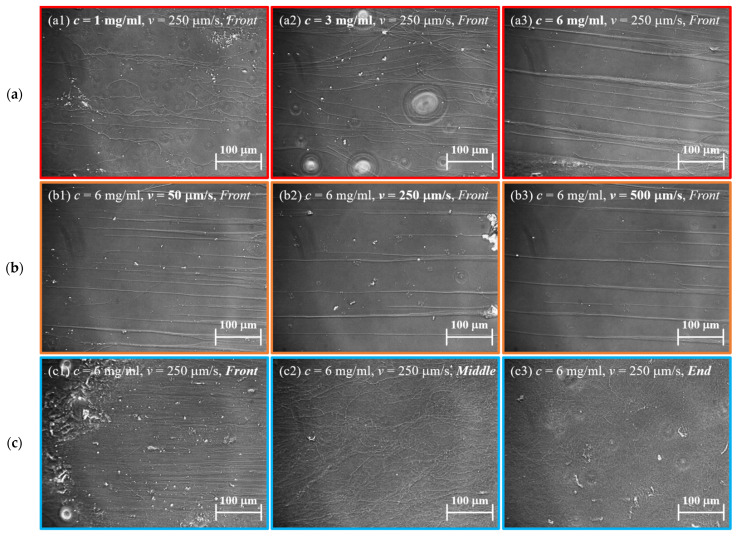
Changes in the morphology and distribution of collagen fibers with the increase in (**a**) concentration from (a1) 1 mg/mL, (a2) 3 mg/mL to (a3) 6 mg/mL; (**b**) stretching speed from (b1) 50 μm/s; (b2) 250 μm/s to (b3) 500 μm/s, and (**c**) stretching position at (c1) Front, in the (c2) Middle, at (c3) End.

The plate tensile loading is achieved by using the entire cover glass slide as the pull plate, with the pull plate moving in a direction parallel to the base surface and bonded to the bottom of the rectangular iron block. During the experiment, the device’s working platform drives the movement of the rectangular iron block via the pull rod, resulting in tensile loading of the collagen fiber. On the one hand, the rectangular iron block acts as a force transfer device, while on the other, it ensures that the pull plate cover slide is as close to the base cover slide as possible, reducing the influence of siphon on the experimental results. The experiment used a collagen fiber solution of 3 mg/mL, a stretching speed of 25 μm/s, and a stretching distance of 20 mm. Figure 3a depicts a significant relationship between fiber orientation and initial fiber solution concentration. The degree of fiber orientation increases as the initial solution concentration increases. Stretching also promotes the aggregation of fibers into larger diameter bundles. When drawing the fiber solution at 1 mg/mL concentration, the protruding fibers cannot gather into bundles, and the straightness of the fibers is the lowest; when drawing the fiber solution at 3 mg/mL concentration, the fibers tend to gather, and the contraction cone angle appears between the fibers, but the fibers cannot gather into tight large-diameter fiber bundles. The fibers stretched out from the 6 mg/mL fiber solution can be aggregated into dense and sturdy fiber bundles with a maximum fiber diameter of 40 μm and the internal of fiber bundles are arranged in parallel. The fiber bundle’s interior is made up of fibers of various diameters (300 nm to 1 μm), and the straightness of small diameter fibers is relatively low. Figure 3b demonstrates that there is no clear relationship between fiber orientation and stretching speed. The length of the oriented fiber bundle, on the other hand, is proportional to the stretching speed. At 500 μm/s stretching speed, the maximum length of a continuous oriented fiber bundle is 2.5 mm, and at 50 μm/s stretching speed, the maximum length of a continuous fiber bundle is 1 mm. Furthermore, as shown in Figure 3c, the number of fibers decreases gradually as the stretching distance increases, while the degree of orientation of the collagen fibers increases. The highly oriented fibers appeared at the end of the solution spreading but did not exhibit the fiber clustering phenomenon. There is no visible fiber in the boundary area between the highly oriented fiber bundle region and the non-fiber bundle highly oriented fiber region.

### 3.2. Observation of Collagen Fibers by SEM and TEM

**Figure 4:**
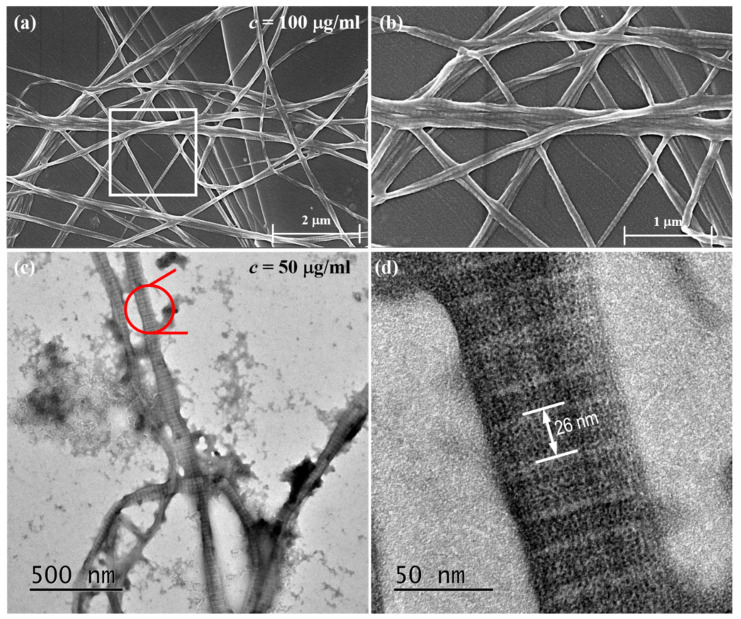
Collagen fibers observed under (**a**) SEM; (**b**) high resolution SEM image showing more morphological details of the regions indicated with square in (**a**); (**c**) TEM image; (**d**) high resolution TEM image showing collagen fiber with D-band of 26 nm as indicated in red circle in (**c**).

The SEM and TEM images of collagen fibers are shown below. When the collagen concentration is 0.1 mg/mL, the collagen fibers are visible under SEM in Figure 4a,b. Due to the force produced by Muscovite’s lattice arrangement [29], some small and oriented collagen fibers can be seen beneath the large collagen fibers. This enables collagen fibers to be arranged directionally. The collagen fibers formed on this basis, on the other hand, are unaffected by the mica substrate, resulting in kink, different diameters, disorderly distribution, and poor orientation. The TEM image is shown in Figure 4c,d, and it clearly demonstrates the D-band characteristics of collagen fibers.

### 3.3. Observation of Collagen Fibers under AFM

**Figure 5:**
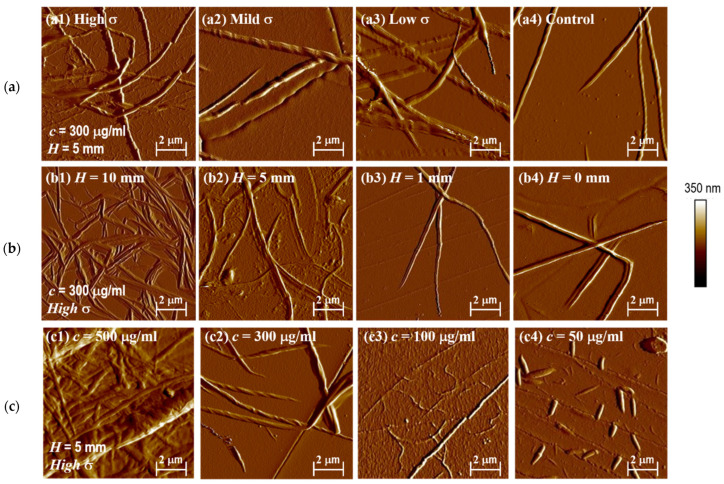
The morphology and distribution of collagen with the change of (**a**) stress-(a1) High stress, (a2) Mild stress, (a3) Low stress, (a4) Control group; (**b**) loading height indicating loading strength (b1) 10 mm, (b2) 5 mm, (b3) 1 mm, (b4) Control group; and (**c**) collagen concentration (c1) 500 μg/mL, (c2) 300 μg/mL, (c3) 100 μg/mL, (c4) 50 μg/mL.

When the collagen concentration was 0.3 mg/mL and the loading height was 10 mm, 5 mL collagen solution was added to the loader and 2.2 mL collagen solution was added to the 35 mm^2^ Petri dish with the same thickness of PDMS film as in the control experiment. This ensures that the liquid height is the same. The stress is greatest in the membrane’s center, resulting in the formation of a large number of collagen fibers (Figure 5(a1)). The number of collagen fibers is low in the middle position (Figure 5(a2)) and it increases in the bottom position, where there are no small collagen fibers (Figure 5(a3)). Gravity deposition could explain the increase in collagen fibers at the bottom. The number of collagen fibers was lowest in the control experiment (Figure 5(a4)). In general, the collagen fibers on the mechanical loader’s culture membrane exhibited more-less-more characteristics. The collagen monomer was subjected to the greatest force and the greatest number of collagen fibers at the center of the membrane. Similarly, collagen fiber diameter was greatest in the center of the membrane, larger at the bottom and small in the middle, and collagen fiber height was greatest in the control group.

### 3.4. Effect of Loading Height of Mechanical Loader on Collagen Self-Assembly

To investigate the effect of force on collagen monomer aggregation further, the stress was varied by varying the loading height of the mechanical loader. The experimental group received 10 mL of solution, while the control group received 3 mL of collagen solution to ensure that the film is under the liquid at different heights, which is used in subsequent experiments.

The collagen fibers in the membrane’s center decrease with decreasing height, as shown in Figure 5. When the height is 10 mm, the most collagen fibers appear (Figure 5(b1)). When the loading height is 5 mm, the number of collagen fibers is more (Figure 5(b2)). The collagen content in the loading height of 1 mm and the control test was lower (Figure 5(b3,b4)). Image J was used to calculate the number of collagen fibers on the culture membrane of the mechanical loader at various heights, and all statistical data is tested using *t*-text at *p* < 0.05. When the loading height was 10 mm and 5 mm, the number of collagen fibers in the high, middle, and low positions showed a more-less-more change rule. There was no significant difference between the middle and low positions. As a result, the number of collagen fibers may be proportional to the amount of stress. The height and diameter of collagen fibers decrease as the loading height decreases.

### 3.5. Effect of Concentration on Collagen Self-Assembly

We set up different collagen solutions for experiments to explore if this rule is concentration dependent. Figure 5c depicts the distribution of collagen fibers in the membrane’s center. The number of collagen fibers decreases as collagen concentration decreases. When the concentration was 0.5 mg/mL, the most collagen fibers were formed. The number of large-diameter collagen fibers gradually decreased as the concentration from 0.5 mg/mL to 0.05 mg/mL, while the number of small-diameter collagen fibers increased. There were only a few scattered collagen fibers and the diameter was smaller when the concentration was 0.05 mg/mL. The large-diameter collagen fibers in the image can be formed by the combination of small collagen fibers in Figure 5(c3). The change in the number of collagen fibers is most visible when the collagen solution concentration is 0.3 mg/mL. There is little difference in the number of collagen fibers between the middle and low positions at 0.5 mg/mL, which may be due to the large number of collagen monomers. Following the formation of the greatest number of collagen fibers in the membrane’s center, there are still enough collagen monomers to form collagen fibers in the middle and deposit in the lowest position. The number of collagen fibers from the center to the edge of the membrane decreased slightly when the collagen solution concentration was 0.1 mg/mL. Except for the central position of the membrane, the number of collagen fibers in the middle and low positions was lower when the collagen concentration was 0.05 mg/mL. This could be due to a lack of collagen monomers and a preference for the formation of collagen fibers at high locations. In the control group, these rules were not obvious. The presence of force makes the difference in the number and shape of collagen fibers in vitro at different concentrations more obvious. As a result, the presence of force can amplify the concentration effect on collagen self-assembly in vitro.

### 3.6. Effect of HA on Collagen Self-Assembly

**Figure 6:**
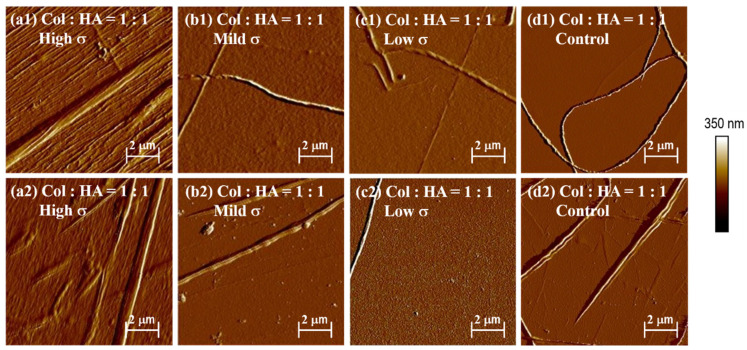
Distribution of collagen fibers in (**a1**,**a2**) high, (**b1**,**b2**) medium, (**c1**,**c3**) low positions, and (**d1**,**d2**) control trial after adding 1:1 HA.

The stress-strain apparatus was used to investigate the effect of HA on collagen self-assembly. The 0.1 mg/mL collagen solution was mixed with a 1:1 ratio of low molecular weight HA solution. The mechanical loader was then used to investigate the effect of different HA proportions on collagen self-assembly in vitro. Figure 6 depicts the experimental results. At the center of the membrane, the addition of low molecular weight HA increases the number and orientation of collagen fibers significantly (Figure 6(a1,a2)). There is no obvious pattern in other places. The addition of HA can increase the height of the collagen fiber layer, as well as the number and diameter of collagen fibers.

## 4. Conclusions

In the plate tensile test, the number of fibers in the solution gradually decreases with distance due to the action of internal friction. The concentration of the solution increases the number of collagen monomers and the number of collagen fibers, causing the collagen fibers to gather into bundles and form large diameter collagen fibers. Furthermore, as the solution concentration increases, so does the viscosity, and the high viscosity solution can firmly adhere to the end of the cover slide, allowing the cover slide to exert an effective stretching effect on the fibers, resulting in the directional arrangement of collagen. The sheer force of the solution, in essence, causes the directional arrangement of collagen fibers. The length of a collagen fiber increases as the stretching rate increases, because the lower the stretching rate, the easier it is to destroy the internal structure of collagen, and the higher the stretching rate, the better the integrity of collagen.

Because the special lattice arrangement of Muscovite exerts a force on the collagen monomer, the force generated by the substrate acts directly on the collagen monomer, resulting in one or more layers of oriented collagen fibers being formed on Muscovite, and the collagen fibers formed on this layer of oriented collagen fibers being clearly visible. These collagen fibers join together to form larger diameter. These collagen fibers will kink because they are formed on the oriented collagen layer and are not affected by the force exerted by the mica substrate during the self-assembly process. Under TEM, the D-band structure of collagen fibers is clearly visible. The D-band structure is a key indicator for identifying collagen fibers. This experiment shows that stress accumulation and gravity deposition coexist during collagen self-assembly on the mechanical loading device, with stress accumulation taking precedence over gravity deposition. The collagen fibers formed in the membrane’s center differed significantly at different concentrations, there was no difference when no force was applied, and the presence of stress magnified the results caused by different concentrations. The amount of collagen in the center of the membrane is proportional to the stress when the loading height and stress are changed, demonstrating the critical role of stress aggregation in the process of collagen self-assembly in vitro. Adequate amount of HA in the mechanical microenvironment can promote the arrangement of collagen fibers along the stress field to resist the effect of external forces. The purpose of this experiment is to explore collagen self-assembly and AFM high resolution mode in a mechanical microenvironment. The goal is to study the morphology, mechanical response, and biological function of collagen using AFM. However, this is only the beginning of the exploration process, and future research should delve deeper into the following areas: (1) To further investigate the mechanical and biological response of collagen under different physicochemical and mechanical conditions, an AFM probe was used to measure the elasticity and viscoelasticity of collagen with different concentrations, hardness substrates, stresses, proportions, and molecular weights of HA. (2) Experiment with different mechanical loading conditions, such as unidirectional tension, compound force tension, and different frequency tension, to see how they affect collagen fiber self-assembly. The load-bearing size of a single collagen fiber and the structural change of collagen fiber during tensile failure were investigated using a micro-stretching device.

## Data Availability

The data presented in this study are available on request from the corresponding author.
